# Tau_i_, A high-resolution metabolic imaging biomarker for myocardium

**DOI:** 10.1186/1532-429X-16-S1-O111

**Published:** 2014-01-16

**Authors:** Charles S Springer, Craig S Broberg, William D Rooney

**Affiliations:** 1 Advanced Imaging Research Center, Oregon Health & Science University, Portland, Oregon, USA; 2 Division of Cardiovascular Medicine, Oregon Health & Science University, Portland, Oregon, USA; 3 Advanced Imaging Research Center, Oregon Health & Science University, Portland, Oregon, USA

## Background

Contrast-enhanced ^1^H_2_O T_1_-weighted cardiovascular MRI is usually interpreted using tracer paradigms; e.g., the extra-/intravascular contrast agent (CA) partition coefficient. However, the signal molecule is water, not CA. Consequently, the myocardial extracellular volume fraction (ECV) is underestimated in proportion to its magnitude. Even more importantly, intercompartmental water exchange kinetics are inaccessible. The mean intracellular water molecule lifetime [tau_i_] is assumed effectively 0; though it is a fraction of a second. For cylindrical myocytes with mean cytolemmal water permeability coefficient P_W _and diameter d: tau_i_^-1^= 4(P_W_/d). tau_i_^-1 ^is linearly related to P_W _and to d^-1^. However, P_W _dominates and is itself dominated by active trans-membrane water cycling. Thus, tau_i_^-1 ^is proportional to the driving cytolemmal ATPase ion pump activity.

## Methods

We acquired serial 1.5T T_1_-weighted ^1^H_2_O data from 6 normal human subjects before and after a single bolus 0.15 mmol/kg CA IV injection. The tissue and blood ROIs comprised ~300 LV wall and ~25 LV voxels [(2 × 2 × 8) mm^3^]. Hematocrit values allowed R_1p _estimation.

## Results

**Figure 1:**
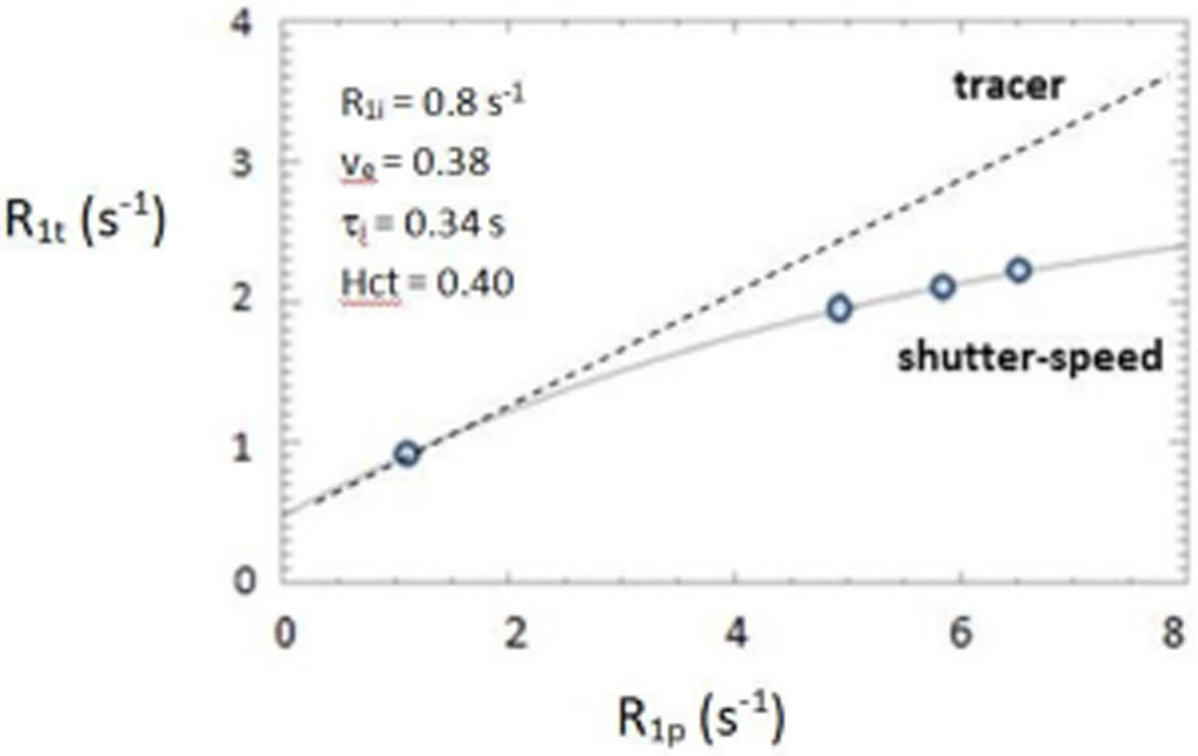
**SSP fitting of DCE-MRI data collected from normal human heart in vivo**. The points represent data collected at four times: one prior to CA administration, and three post-CA administration. R_1t _is the myocardial tissue ^1^H_2_O R_1 _value, and R_1p _is the blood plasma ^1^H_2_O R_1 _value calculated for a hematocrit (Hct) of 0.4. The solid curve represents the best SS model fitting to the data with parameters shown in the inset. The dashed asymptotic line is expected for the tracer paradigm.

Figure 1 plots ROI ^1^H_2_O LV wall tissue (R_1t_) vs. corresponding LV plasma (R_1p_) values during the bolus passage [R_1 _≡ T_1_^-1^]: 3 post-CA points and 1 pre-CA, for one subject. These are fitted with a shutter-speed (SS) two-site-exchange [2SX] expression approximating CA extravasation steady-state, [CA_o_] = [CA_p_] (o, interstitial); the solid curve [only v_e _and tau_i _varied]. The extracellular volume fraction v_e_(SS) [≡ ECV(SS)] is 0.38. The tracer paradigm (TP) predicts a straight line for the R_1t _R_1p_-dependence, with slope v_e_: the dashed asymptote. In order to fit the non-linear data, the TP straight line must be pivoted down about the origin: this yields ECV(TP) = 0.25, a 34% reduction. SS success is not a fitting goodness issue: the TP line through the data incurs residuals scarcely larger than for the 2SX curve. Crucial is the systematic TP ECV depression, which increases in pathology. Even more important is the SS access to tau_i _- because of the active trans membrane water cycling link to metabolic activity. We obtain tau_i _= 0.34 s for this subject, the first reported for human myocardium [means in Table 1]. (Since [CA_o_] > [CA_p_], v_e _and tau_i _are over- and underestimated.) Pixel by-pixel v_e _and tau_i _values allow parametric mapping.

**Table 1:** [n = 6]

ECV(TP)	0.26 (+/- 0.02)
ECV(SS)	0.33 (+/- 0.04)
tau_i_	0.20 (+/- 0.09) s

## Conclusions

The first tau_i _metabolic sensitivity hint came in a 2006 perfused ex vivo rat heart study (with other collaborators) finding that (no flow) ischemia increased tau_i _by 56% - from 0.18 to 0.28 s. For control mice tau_i _= 0.19 s, and for a hypertensive mouse model tau_i _= 0.44 s, values have been reported. The very large (132%) tau_i _increase is accompanied by an only 30% d increase; from 20 to 26 μm. These results demonstrate P_W _dominance of tau_i_, and sensitivity to metabolic activity slowing caused by both ischemia and chronic hypertension.

## Funding

NIH [RO1 NS40801].

